# Supplementary material for: The SpinBus architecture for scaling spin qubits with electron shuttling
Source: Nat Commun. 2024 Jun 11;15:4977. doi: 10.1038/s41467-024-49182-4 (PMC11166970; doi:10.1038/s41467-024-49182-4)
Supplement: Supplementary file 1 — Supplementary Information PDF file [file 41467_2024_49182_MOESM1_ESM.pdf]

The SpinBus architecture for scaling spin qubits with electron shuttling

Matthias Künne[1†], Alexander Willmes[1†], Max Oberländer[1], Christian Gorjaew[1], Julian D. Teske[1], Harsh Bhardwaj[1], Max Beer[1], Eugen Kammerloher[1], René Otten[1,2], Inga Seidler[1], Ran Xue[1], Lars R. Schreiber[1,2*], Hendrik Bluhm[1,2*]

[1]JARA-FIT Institute for Quantum Information, Forschungszentrum Jülich GmbH and RWTH Aachen University, 52074 Aachen, Germany.
[2]ARQUE Systems GmbH, 52074 Aachen, Germany.

*Corresponding author(s). E-mail(s): lars.schreiber@physik.rwth-aachen.de; bluhm@physik.rwth-aachen.de;
[†]These authors contributed equally to this work.

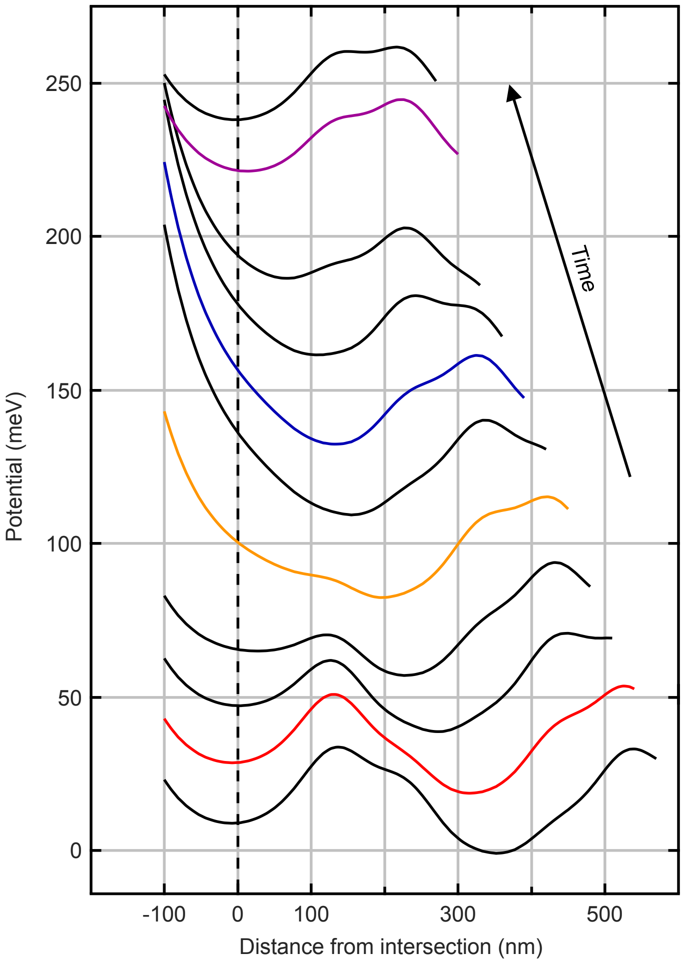

Supplementary Figure 1: **Potential line cuts during T-junction operation**. Line cuts of the potential for different times during corner shuttling. Colors correspond to the frames in Fig. **2**b of the main text. Successive line cuts are shifted for clarity. At any point in time during corner shuttling, sufficient confinement can be maintained.

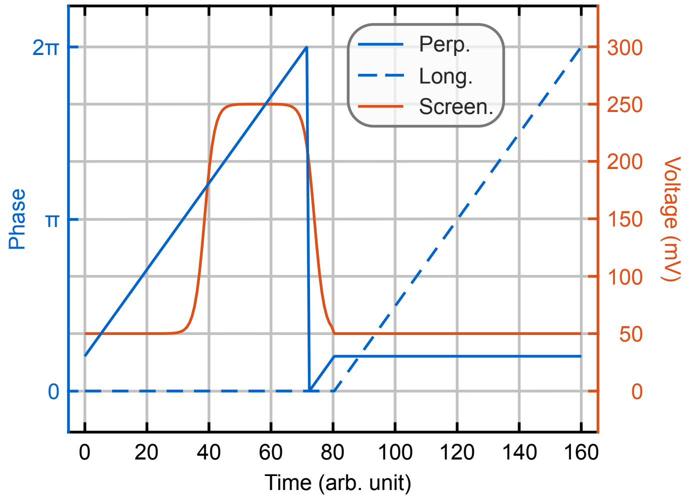

Supplementary Figure 2: **Shuttling phases during T-junction operation**. Time evolution of the shuttling phases during corner shuttling for the initial perpendicular (solid blue line) and target longitudinal (dashed blue line) QuBus device, respectively. The voltage applied to the continuous screening gate is dynamically adjusted (orange line) to ensure an orbital splitting within the target range.

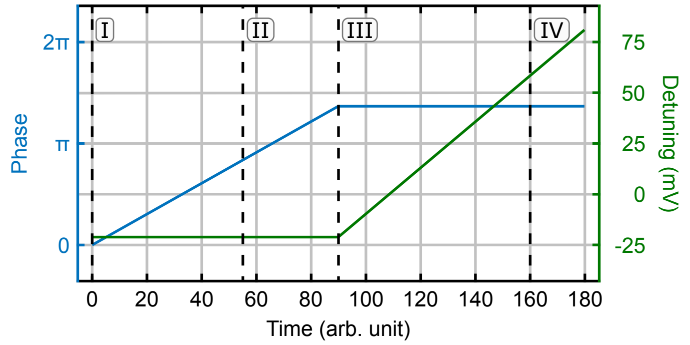

Supplementary Figure 3: **Time evolution of the shuttling phase $\varphi(t)$ and inter-dot-detuning in the IR zone during initialization.** Dashed lines indicate the points in time of the potential line cuts in Fig. **3**c of the main text.

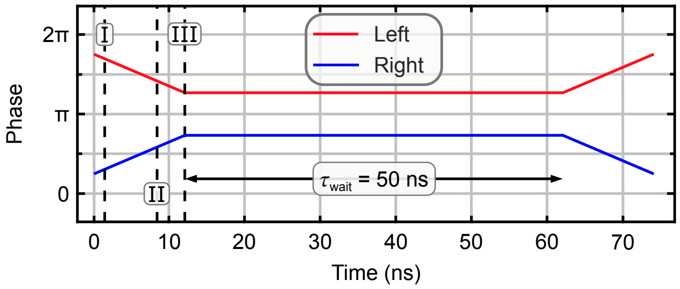

Supplementary Figure 4: **Time evolution of shuttling phases in the manipulation zone during a two-qubit operation.** Included is a wait time of $\tau_{\text{wait}} = 50\,\text{ns}$ for the actual two-qubit gate. Dashed lines indicate the points in time of the potential line cuts in Fig. **4**c of the main text.

Supplementary Note 1: Dealing with random valley splitting due to alloy disorder

An important aspect for the realization of any shuttling-based architecture in the Si/SiGe platform will likely be the distribution of the variation of the valley splitting in Si/SiGe heterostructures. A recent publication by Wuetz *et al.*[1] suggests that the valley splitting is dominated by alloy disorder and thus varies randomly across different quantum dot positions. Occasional small valley splittings are unfavorable for resonant EDSR-driving with fast electron movement and shuttling coherence, but can be mitigated by adjusting the shuttling path and manipulation position. The correlation length of the valley landscape is expected to be given by the spread of the electron's wave functions ($\approx 20\,\mathrm{nm}$), consistent with recent experiments[2] that probe the valley splitting along a shuttling device. For the manipulation, simply stopping at a slightly different location in the roughly 200 nm wide region of favorable magnetic field gradient (see Fig. **4**b) is thus very likely to avoid a problematic spot. For the proposed operating mode, valley-related single-qubit errors including their mitigation are discussed in detail in Ref.[3]. The shuttling path can be shifted laterally by applying voltages to the screening gates laterally defining the channel. A detailed study in Ref.[4] shows that, potentially in combination with slowing down by a factor three to five in remaining problematic spots, this can lead to transfer fidelities of 99.9% and higher over 10 µm with a very high probability. The voltage changes can be applied dynamically in synchronization with the electron motion. Alternatively, the screening gates can be split into individual, roughly micron-long sections to which different DC voltages are applied. The latter approach trades dynamical signals in favor of a larger number of DC voltages. In addition, possible modifications of the heterostructure, including Ge spikes[5], increased[1] or oscillating[6] Ge concentrations inside the quantum well, may reduce the occurrence of small valley splittings or ideally avoid them altogether. In the worst case, a faulty unit cell can be omitted by mapping an algorithm to physical qubits differently.

Supplementary Note 2: Wiring complexity and solutions

As the wiring density can be a main limiting factor for the size of processor achievable with a given integration approach, we present an estimate of the number of required signals based on an economical operating strategy. The shuttling lanes in Fig. **1**c and d are color-coded accordingly to indicate which shuttling lanes are controlled globally or at the individual unit cell level. The vertical shuttling lanes colored green are intended for the connection of unit cells and are controlled at a global level, so that four shared AC signals can suffice. The envisioned operating mode is that these shared lanes periodically move up and down by one or several unit cell sizes and that electrons can be transferred onto them from the horizontal red and blue lanes, which are controlled individually for each unit cell. Either of these individually controlled shuttling lanes is also used for single-qubit gates, while both of them are utilized for a two-qubit gate at their junction. The use of the same signals for the vertical and horizontal red sections in one unit cell is enabled by the assumption that the IR zone is only used when no qubit in the horizontal red lane and no qubit in the blue lane is near the T-junction to the IR zone when the red horizontal lane is active. For the independent operation of the IR zones and to compensate disorder, we also propose local control of the first two individual clavier

gates in the shuttling lane and of the SET plunger gate to maintain a sensitive operating point. In addition, one readout wire per cell needs to have a Megahertz-scale bandwidth. To ensure a sufficiently large orbital splitting at all times during corner shuttling, we propose connecting an individual AC signal to the screening gate segment of each T-junction to allow for dynamic adjustment of the confinement. Using a separate DC signal instead may ultimately turn out to be sufficient, tough. If the shuttling path needs to be controlled to avoid disorder or sites with low valley splitting (see Supplementary Note 1), a few signals per unit cell applied to the screening gates need to be added, with the exact number depending on the required degree of independent control. We thus estimate that a quantum processor chip with N unit cells requires $15N+4$ AC signals.

Analogously, DC voltages are also either shared across all unit cells or individually provided for each unit cell. For the screening gates defining the shuttling channels, inducing gates for SET reservoirs and the global top gate, three global DC voltages are sufficient. For adjusting the reservoir potential to the shuttling lanes, the voltage on the second Ohmic contact to the SET should be tunable. In addition, two SET barrier gates must be tuned. T-junctions and manipulation zones do not require additional DC voltages. Lastly, grounding of the micromagnets is optional and can be shared across all micromagnets. Hence, $3N+4$ DC voltages are needed.

While there are no inherent scaling limitations beyond gate and shuttling errors to our architecture at the quantum layer, the wiring requirements have to be compatible with cryostat wiring, packaging and back-end-of-line (BEOL) technology. We estimate that currently available wiring solutions in cryostats of about 1,000 coaxial cables[7] are the main limiting factor and can accommodate a quantum processor chip with $9\times 8=72$ unit cells. The highest on-chip wiring density occurs at the edge of the quantum plane and requires a few wiring layers for fan-out, which can be realized using standard BEOL technology. Assuming a unit cell size of $10\,\mu\text{m}\times 10\,\mu\text{m}$ and a conservative wiring pitch of 500 nm in the on-chip wiring layers, each wiring layer can accommodate 720 lanes along the circumference of the quantum layer, whereas unit cells provide sufficient space to extend all vias across the required number of wiring layers. Thus, a few wiring layers are sufficient to route all signals to the edges of the quantum processor chip or spread them across the surface depending on the connection scheme.

For connecting the chip to a printed circuit board (PCB), high-density socket solutions are an established technology enabling as high as 6,096 connections in consumer devices[8]. Commercial land grid array (LGA) solutions that allow for chip-to-board connection are available with contact pitches as low as 0.4 mm and are validated for cryogenic operation[9]. Assuming a dense connection arrangement and neglecting additional ground connections this results in a chip size of 14 mm by 14 mm. Finally, we estimate the footprint of the connection from the PCB to the coaxial wiring in the cryostat based on current sub-miniature coaxial connectors. For example, Rosenberger WSMP® connectors can feature a center-to-center spacing of 2.15 mm while maintaining the signal integrity of a coaxial connector for frequencies up to 100 GHz[10]. This results in a PCB area of $\sim 50\,\text{cm}^2$ for 1000 connectors, including some overhead for screws etc. Flex-cable solutions can enable an even higher density[11]. DC wiring using flex-connectors will have a negligible footprint compared to the high frequency connections.

Supplementary References

[1] Wuetz, B. P. *et al.* Atomic fluctuations lifting the energy degeneracy in Si/SiGe quantum dots. *Nat. Commun.* **13**, 7730 (2022).

[2] Volmer, M. *et al.* Mapping of valley-splitting by conveyor-mode spin-coherent electron shuttling. Preprint at https://arxiv.org/abs/2312.17694 (2023).

[3] Pazhedath, A. M. *et al.* Large spin shuttling oscillations enabling high-fidelity single qubit gates. Preprint at https://arxiv.org/abs/2403.00601 (2024).

[4] Losert, M. P. *et al.* Strategies for enhancing spin-shuttling fidelities in Si/SiGe quantum wells with random-alloy disorder. Preprint at https://arxiv.org/abs/2405.01832 (2024).

[5] McJunkin, T. *et al.* Valley splittings in Si/SiGe quantum dots with a germanium spike in the silicon well. *Phys. Rev. B* **104**, 085406 (2021).

[6] McJunkin, T. *et al.* SiGe quantum wells with oscillating Ge concentrations for quantum dot qubits. *Nat. Commun.* **13**, 1–7 (2022).

[7] Bluefors XLDsl Dilution Refrigerator System. https://bluefors.com/products/xldsl-dilution-refrigerator (Accessed: 2024-05-05).

[8] AMD EPYC™ 9654. https://www.amd.com/en/products/cpu/amd-epyc-9654 (Accessed: 2024-05-05).

[9] Ardent Concepts High Density TR Multicoax™ Cabling. https://www.ardentconcepts.com/quantum-overview (Accessed: 2024-05-05).

[10] Rosenberger WSMP® NEW GENERATION connectors. https://www.rosenberger.com/product/wsmp/ (Accessed: 2024-05-05).

[11] Cummings, J. D., Rokosz, J. A., Thompson, K. J. & Weber, S. J. High-Density Cryogenic Wiring for Superconducting Qubit Control. Patent US20220230785A1 (2022).
